# Sex hormone allergy: clinical aspects, causes and therapeutic strategies – Update and secondary publication

**DOI:** 10.1186/s40413-017-0176-x

**Published:** 2017-12-27

**Authors:** E. Untersmayr, A. N. Jensen, K. Walch

**Affiliations:** 1 Institute for Pathophysiology and Allergy Research, Center for Pathophysiology, Infectiology and Immunology, Medical University of Vienna, Waehringer Guertel 18-20, 1090 Vienna, Austria; 2 AllergyCare – Allergy Diagnosis and Study Center Vienna, Vienna, Austria; 3 Department of Gynecological Endocrinology and Reproductive Medicine, Clinic of Obstetrics and Gynecology, Medical University of Vienna, Vienna, Austria

**Keywords:** Allergy, Sex hormone, Progesterone, Estrogen, Female, Sex, Gender, Pregnancy, Pregnancy loss, Transgender, Desensitization

## Abstract

Sex hormone allergy as a clinical syndrome has been known for almost a century. Due to the diversity of clinical presentation regarding symptoms and disease patterns, the optimal patient care represents an enormous interdisciplinary challenge. Frequently, hypersensitivity reactions affect more than one sex hormone and double positive tests for estrogen and progesterone have been described. Since the menstrual cycle dependent symptoms range from skin afflictions, gynecological problems to non-specific reactions, different pathophysiological mechanisms seem likely.

Various desensitization protocols are described as causal treatment options, but are rarely applied in clinical routine. Consequently, major research efforts with a quick translation of therapeutic interventions into clinical practice will be crucial to help affected patients in the future.

## Background

Sex hormones not only influence the female or male phenotype, they also substantially contribute to the development and regulation of numerous physiological processes within the human body. Due to worldwide research efforts, the knowledge of cellular distribution patterns of different steroid-hormone receptors is steadily increasing [1–4]. Hence, the complex connections between sex hormones and organ development are better understood today [5]. Sex hormones are not only involved in the development and function of sex organs, but also have a great influence on neuronal or pulmonary structure and function [6–8]. This knowledge contributes to the concept of gender dimorphisms in the context of the physiological embryonic and infantile development, regarding neurological and psychiatric sex differences as well as development of pathologies being of high importance for optimal patient care [6–8].

Via their respective receptors, sex hormones influence the function and activity of immune cells shaping inter-individual differences in defense against diseases and in formation of allergies and autoimmune disorders in patients [4, 9].

### Hormone allergy – A paradox of nature

Besides the fundamental impact of sex hormones on the human body, steroid hormones can trigger what is still a rarely diagnosed disease, which is  hormone allergy. For nearly a century different research groups across the world have documented a connection between menstrual cycle related complaints in women and immunologically mediated hypersensitivity to sex hormones. Menstrual cycle dependent urticaria and hypersensitivity reactions to sex hormones were first reported as early as 1921. In this first published case study pre-menstrually collected, autologous patient’s serum was injected intravenously confirming its role as trigger for the observed skin reactions [10]. After this first published report, further scientific evaluations followed and soon the term "hormone allergy" was coined [11–13]. Moreover, even to date the autologous serum skin test is performed as a screening test for autoantibodies in the extended diagnostic program of patients with chronic spontaneous urticaria [14]. Additionally, questions regarding an association between menstrual cycle and chronic urticaria were suggested to be included for obtaining a detailed clinical history as a first step of urticaria diagnosis [14].

In 2004 a study pinpointed the relation between premenstrual syndrome (PMS) with or without concurrent skin symptoms such as pruritus vulvae, hyperpigmentation or acne vulgaris and sensitization against estrogen and/or progesterone diagnosed via intradermal testing for the first time [15]. Immediate type or delayed type hypersensitivity reactions were observed for all 20 included patients with the above described clinical symptoms [15]. Ten healthy controls did not reveal any hypersensitivity reactions upon intradermal testing with sex hormones.

Shortly thereafter another study compared estrogen or progesterone specific antibody levels in blood samples of patients with menstrual cycle related complaints such as asthma, migraine or joint pains to antibody levels measured in a healthy control group [16]. Higher levels of estrogen and progesterone specific IgG, IgM and IgE antibodies were determined in the patient cohort with menstruation cycle dependent disorders. With regards to this study, a possible mechanistic role of the polyclonal antibody response with various immunoglobulin isotypes has to be taken into consideration as it is observed also for other autoimmune disorders [17]. Thus, also cytotoxic antibodies and/or effector cell activation might trigger adverse reactions.

Other studies reported a correlation between habitual idiopathic pregnancy loss and local sex hormone hypersensitivity reactions diagnosed by positive intradermal skin reactions towards estrogen and progesterone [18, 19]. Immediate type hypersensitivity reaction was assessed in one study revealing more than 50% of patients having a positive skin test reaction at 20 min [18]. Both studies evaluated delayed type hypersensitivity reactions against estrogen or progesterone at 24 h with positive tests in approximately 70% of patients in the cohort with repeated miscarriage during early pregnancy (Table 1) [18, 19]. In both studies a small subgroup of 15% of the patients did not show any skin reactivity to the tested steroid hormones. Of interest, other autoimmune triggers such as cytotoxic antibodies, non-suitable human leukocyte antigens, deviated natural killer (NK) cell function and distribution were previously reported for habitual miscarriages [20–22]. Moreover, patients with estrogen-mediated dermatitis also have altered levels of other sex hormones. One study reported that the levels of testosterone and luteinizing hormone were significantly higher in 14 estrogen-sensitized patients compared to the healthy control group while progesterone-levels were significantly lower [23]. Despite this correlation between miscarriage and hormone hypersensitivity, testing for hormone allergy is not included in the recommendations in current guidelines for systematic follow-up of patients with recurrent pregnancy loss [24].

**Table 1:** Local hypersensitivity reactions upon intradermal skin testing with estrogen and progesterone in patients with early recurrent pregnancy loss compared to healthy controls [18, 19]

Study	*Itsekson AM* et al. *Am J Reprod Immunol. 2007* [19]		*Ellaithy MI* et al. *J Reprod Immunol. 2013* [18]	
	patients	controls	patients	controls
Estrogen hypersensitivity	23	0	32	0
Progesterone hypersensitivity	20	0	34	0
Combined hypersensitivity	17	0	26	0
No hypersensitivity	3	10	7	12
Total numbers patients/control	29	10	47	12

### Clinical presentation of hormone allergy

As outlined above there is a link between cyclic symptoms like PMS, menstrual cycle dependent asthma, headaches and joint pain as well as recurrent miscarriages and steroid hormone allergy. Hypersensitivity to steroidal sex hormones, however, can also be associated with several other clinical manifestations such as dermatitis, dysmenorrhea, rhinitis, itching and bullous erythema multiforme. Additionally, psychological disorders have been described [25]. Sensitization against sex hormones has been discussed as a possible cause for hyperemesis gravidarum, infertility and premature births [25–28].

**Fig. 1:**
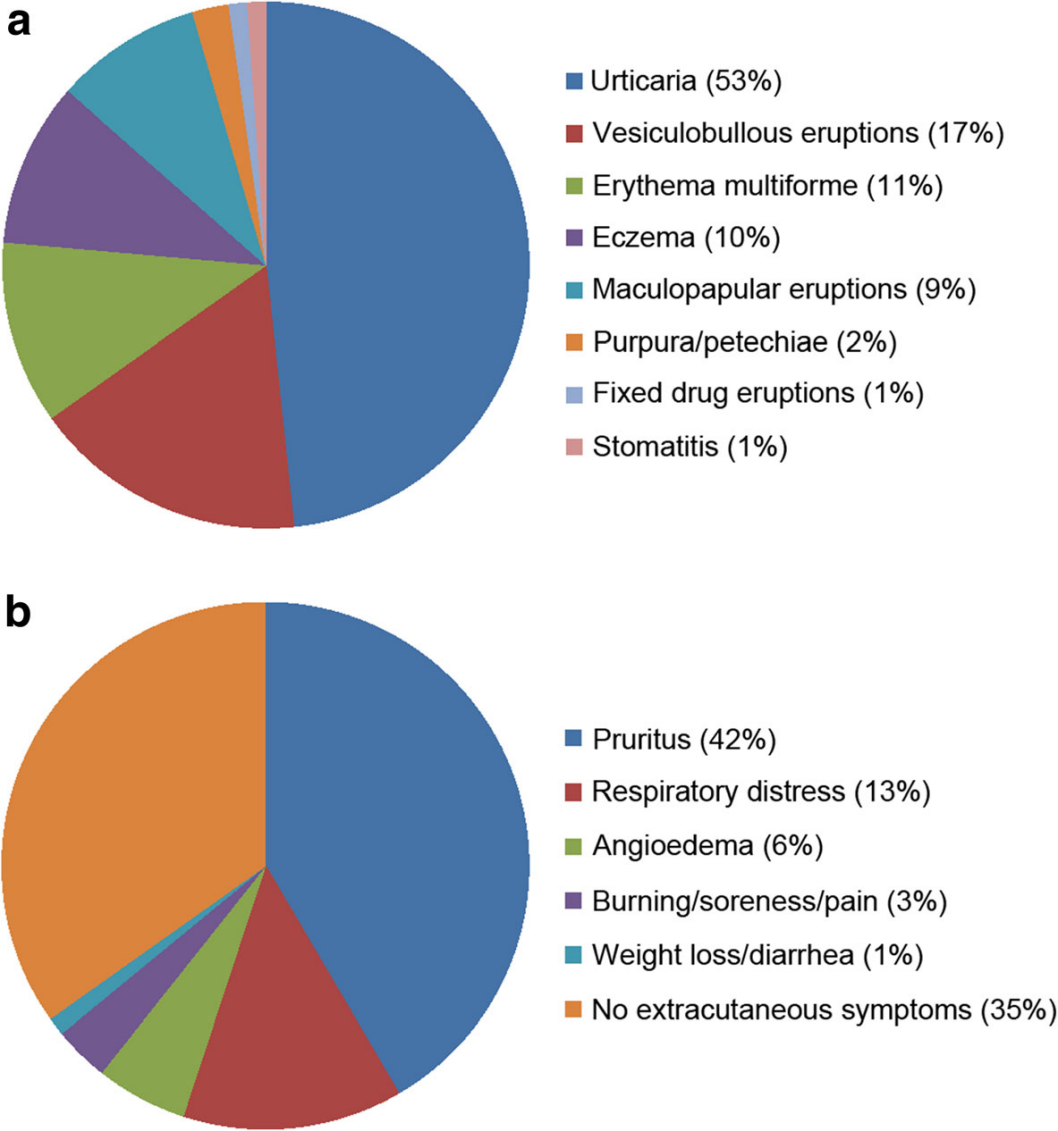
Frequency of different clinical manifestations of autoimmune progesterone dermatitis. Progesterone dermatitis is a rare disease with a broad spectrum of symptoms triggered by hypersensitivity reactions to the endogenous or exogenous steroid hormone. Based on available literature regarding clinical presentation a recent review article summarized the variety of cutaneous symptoms (**a**) as well as associated disease manifestations during each flare-up including extracutaneous symptoms (**b**) observed in 89 patients with progesterone dermatitis [32]. The percentage of patients with each clinical presentation as described by Nguyen and colleagues [32] is given in parenthesis

For decades the skin has been accepted as the primarily affected organ, with the main diagnoses for sex hormone skin affections being estrogen or progesterone dermatitis. The possible dermal manifestations range from itching, urticaria, eczema, papillo-vesicular or vesiculobullous dermatosis, erythema multiforme, hirsutism with or without acne and hyperpigmentation, purpura and petechiae to stomatitis [23, 29–31]. In accordance with this large variety of different symptoms, a recent review on currently available scientific data concerning autoimmune progesterone dermatitis focused on the broad spectrum of clinical disease presentation (Fig. 1) and almost half of the evaluated patients showed a generalized involvement of three or more areas of the body [32]. Based on the diversity of symptoms, the authors’ conclusion on different possible pathophysiological mechanisms seems logical, which will have to be confirmed and defined by future research efforts in the field.

In severe cases, sex hormone allergies can even lead to anaphylaxis, a potentially life-threatening allergic reaction with rapid onset [33]. Case studies showed that patients suffer from unexplained anaphylactic reactions for years before being adequately diagnosed with sex hormone allergy [34–38].

Of interest, the onset of hormone associated complaints is not solely associated with menarche and the monthly hormonal fluctuations thereafter. The development of hormone hypersensitivity has also been linked to pregnancy, intake of exogenous estrogen or progesterone, oral contraception pills and in vitro fertilization procedures. Again, these different possibilities for disease onset and progression points towards multiple possible causes, such as exogenous hormone administration, raising hormone levels during pregnancy or hormone cross sensitivity [32, 39].

### Potential causes for hormone allergy

The exact pathophysiological mechanisms leading to the development of hormone allergies have not been elucidated to date. However, in scientific literature similar mechanisms as those being associated with drug allergies, i.e,. the immunological response to drug compounds [40], have been described. Thus, the pathophysiological role of IgE antibodies, T-cells, dendritic cells as well as abnormal cytokine or NK cell responses is currently under discussion [25, 41]. Depending on underlying mechanism, the resulting clinical pictures might vary [32]. It seems logical to suspect an IgE-mediated process as causal for a fast appearing urticarial reaction. Eczematous reactions on the other hand might indicate T-cells as primary effector cells.

The cause behind steroid hormone hypersensitivity still remains unclear to date. The intake of xenoestrogens and endocrine disruptors like estradiol valerate, atrazine and bisphenol A as well as the use of oral contraceptives has been discussed as potential triggers of disease [25]. Accordingly, the rate of exogenous sex hormone medication in the medical history of patients with sex hormone allergy is high and immunological mechanisms like uptake of exogenous hormones by antigen presenting cells and subsequent T cell activation might play a role [32]. Hypersensitivities to oral contraceptives are a known entity and were first described decades ago. Moreover, the potential of transdermal estrogen patches to trigger local allergic reactions is well documented [42–47].

In an experimental rat model for hormone hypersensitivity the synthetic estrogen estradiol valerate was used as an endocrine disruptor and administration of estradiol valerate to the rats resulted in recurrent miscarriage [48]. It seems that the route of estradiol valerate administration influences its efficacy as an endocrine disruptor. When estradiol valerate is absorbed through the skin, the associated presentation to immunocompetent cells seems to increase its potential as an endocrine disruptor [25]. Treatment of transsexual persons with high-dose, frequently administered transdermally, off-label used sex hormones might pose a risk for the development of hormone hypersensitivities, even though allergic complaints are not being reported as possible side effects of these treatments [49–52]. Furthermore, cross-reactions to other steroid hormones like hydrocortisone are under discussion as possible triggers for hormone allergy [39].

### Diagnosis and treatment options for steroid hormone hypersensitivity

The symptoms associated with hormone allergies are severe and the impact of recurrent pregnancy loss on psychological and physical health is great. Thus, offering a suitable diagnostic concept and possible therapeutic options is essential for adequate patient care. Due to a lack of validated laboratory tests, a detailed patients history and the timely correlation of symptoms with cyclic hormone fluctuations still play a major role [25]. Another important part of the final diagnosis is the intradermal testing with 0.02 mg of the possible triggering hormones during the luteal phase of the menstrual cycle. Simultaneous intradermal application of only the carrier substances provides a reliable control. The reading of the results takes place after 20 min, 24 h, 48 h and 7 days [18, 19, 27, 53].

Different treatment strategies with a thorough assessment of benefits versus side effects can be considered after an appropriate diagnosis has been made. These therapeutic approaches include systemic corticosteroids, conjugated estrogen, the anti-estrogen *Tamoxifen* and oral contraceptives, as summarized by Nguyen and colleagues [32]. Medication reduces symptoms but is not curative for the disease. The only causative treatment options available to date are different desensitization protocols, which have to be carefully selected based on the patient’s clinical need. The first desensitization approach dates back to the beginning of the last century. Urticarial symptoms of a patient were successfully treated with intradermal application of autologous, pre-menstrually obtained serum [54]. Since then different desensitization protocols have been published in scientific literature, describing oral, intradermal or intravaginal application of the hormones being defined as triggers of allergic symptoms in the patients. The targeted disease patterns range from PMS, dysmenorrhea, hyperemesis gravidarum to enabling in vitro fertilization in autoimmune progesterone dermatitis [15, 25, 26].

Fast desensitization protocols before performing in vitro fertilization have been developed, with the administered hormone dose being increased every 20 min in 8 to 10 steps [27]. Another equally successful desensitization protocol for PMS and habitual miscarriages were three intradermal injections of increasing hormone doses over the course of three months [55]. The most recently published study describes 24 cases of progestogen hypersensitivity with a detailed diagnostic approach determining exogenous or endogenous progestogen sources as triggers of reactions and reporting different routes of desensitization as suitable treatment protocols [56].

Still to this day, the diverse desensitization methods can only be considered as experimental approaches. The correct diagnosis and a careful patient selection must be considered as absolutely essential for the clinical success of any therapeutic intervention.

## Conclusions

The data summarized here underline the urgent need for an interdisciplinary treatment approach for sex hormone allergies. As gynecological and dermatological symptoms are most frequently observed, a broad understanding of the disease and the cooperation of diverse medical disciplines are essential. Since this disease is accompanied by a large variety of symptoms and diverse clinical presentations different pathophysiological mechanisms might be causative. Hence, individual and precise diagnostic approaches are pivotal. Without a doubt, further intensive research efforts will be decisive to detect the cause for disease and to define the optimum therapeutic strategies for sex hormone allergies in the future.

## Abbreviations

Ig: immunoglobulin
PMS: premenstrual syndrome
